# Comparing the Efficacy and Safety of Warfarin Monotherapy vs. Warfarin and Aspirin for Adult Patients With Left Ventricular Assist Devices: A Meta-Analysis

**DOI:** 10.7759/cureus.53101

**Published:** 2024-01-28

**Authors:** Revanth Reddy Bandaru, Anurag Rawat, Illahay Jalali, Abraham K Isaak, Alahed A Alrahahleh, Sohaib M Bataineh, Calvin R Wei, Shamsha Hirani

**Affiliations:** 1 Internal Medicine, East Carolina University, Greenville, USA; 2 Interventional Cardiology, Himalayan Institute of Medical Sciences, Dehradun, IND; 3 Medicine, Tehran University of Medical Sciences, Tehran, IRN; 4 Telemetry, Sharp Memorial Hospital, San Diego, USA; 5 Internal Medicine, Orotta School of Medicine and Dentistry, Asmara, ERI; 6 Medicine, Yarmouk University, Irbid, JOR; 7 Research and Development, Shing Huei Group, Taipei, TWN; 8 Cardiology, Baqai Hospital, Karachi, PAK

**Keywords:** systematic review and meta-analysis, left ventricular assist device:, efficacy, aspirin, warfarin

## Abstract

The aim of this meta-analysis was to assess the safety and efficacy of warfarin plus aspirin versus warfarin monotherapy in patients with left ventricular assist devices (LVAD). The present meta-analysis was conducted using the guidelines of Preferred Reporting Items for Systematic Reviews and Meta-Analyses (PRISMA). Two authors systematically searched online databases, including PubMed, EMBASE, the Cochrane Library, and Web of Science from inception to December 31, 2023. Outcomes assessed in this meta-analysis included any thrombotic event, bleeding events, and all-cause mortality. A total of five articles were included in the meta-analysis, enrolling a pooled sample size of 876 patients, including 405 in the warfarin monotherapy group and 471 in the warfarin plus aspirin group. Pooled analysis showed that the risk of thrombotic events was not significantly different between the two groups (risk ratio (RR): 0.46, 95% confidence interval (CI): 0.15-1.37). The risk of bleeding events was significantly lower in patients receiving warfarin alone compared to patients receiving aspirin plus warfarin (RR: 0.67, 95% CI: 0.53-0.85). The risk of all-cause mortality was not significantly different between patients receiving warfarin alone and patients receiving aspirin plus warfarin (RR: 0.92, 95% CI: 0.65-1.30). Despite the potential benefits of discontinuing aspirin, the decision should be approached cautiously, considering the undefined risks of discontinuing anticoagulation in LVAD patients.

## Introduction and background

Patients experiencing end-stage heart failure who have undergone implantation of a continuous-flow left ventricular assist device (CF-LVAD) exhibit substantial enhancements in both quality of life and survival. As a result, CF-LVADs are now regarded as a preferred option over optimal medical therapy for individuals ineligible for cardiac transplant [1,2]. Despite notable advancements in CF-LVAD technology spanning the past two decades, complications related to bleeding and thrombotic events remain challenging [3]. Hematologic complications, including intracranial hemorrhage, gastrointestinal bleeding, pump thrombosis, and ischemic stroke, frequently occur in mechanical circulatory support involving CF-LVADs [4,5].

The International Society of Heart and Lung Transplantation (ISHLT) Guidelines for Mechanical Circulatory Support advocate the simultaneous use of warfarin and aspirin (ASA) in CF-LVAD patients to mitigate the risk of thrombotic events [6]. Both the American Association for Thoracic Surgery/International Society for Heart and Lung Transplantation (ISHLT) guidelines on selected topics in mechanical circulatory support and the 2019 European Association for Cardio-Thoracic Surgery (EACTS) Expert Consensus on long-term mechanical circulatory support recommend chronic anticoagulation with a vitamin K antagonist (VKA) for patients with a durable CF-LVAD [7,8]. The concomitant use of aspirin is also recommended, albeit with a level of Evidence C, indicating reliance on expert opinion. However, recent years have witnessed growing skepticism regarding the safety and efficacy of aspirin as an adjunct antithrombotic agent to VKA, leading to a more variable usage of aspirin.

The uniformity of antithrombotic therapy among diverse healthcare facilities has been impeded by the development of device-specific international normalized ratio (INR) targets and the prescription of varied doses of ASA, ranging from 81 to 325 mg on a daily basis. Clinicians face a lack of direct evidence comparing different antithrombotic strategies in CF-LVAD patients, making it challenging to choose an empirical regimen for preventing bleeding and thrombosis. Consequently, there is a pressing need for a comprehensive pooled analysis of available studies to assess and compare the safety and efficacy of warfarin plus aspirin versus warfarin alone in patients with left ventricular assist devices.

## Review

Materials and methods

The present meta-analysis was conducted using the guidelines of Preferred Reporting Items for Systematic Reviews and Meta-Analyses (PRISMA). Two authors systematically searched online databases, including PubMed, the Cochrane Library, EMBASE, and Web of Science from inception to December 31, 2023. Keywords used to search for relevant articles included “warfarin monotherapy”, “aspirin”, and “left ventricular assist device”. Medical Subject Heading (MeSH) terms were used along with synonyms. We examined reference lists of all included studies and relevant reviews for additional studies. The exhaustive collection of studies identified in our initial search was imported into Endnote, where duplicate studies were subsequently eliminated.

Eligibility Criteria

Studies were selected based on the PICO criteria. We included cohort (prospective or retrospective) or randomized controlled trial (RCT) studies encompassing adult patients with left ventricular assist devices (participants). Studies compared warfarin monotherapy (exposure) with a combination of warfarin and aspirin (comparison group). We included studies that assessed thrombotic events, bleeding events, and all-cause mortality. We excluded case reports, case series, reviews, studies not including humans, commentaries, and literature reviews. We also excluded studies lacking a comparison group.

Data Extraction and Quality Assessment

Initially, two authors screened the titles and abstracts of all identified articles to determine eligibility. Subsequently, full-text articles were screened based on the initial assessments of titles and abstracts. Discrepancies were resolved through discussion to achieve consensus. In cases where resolution was difficult, a third reviewer was consulted to facilitate consensus. We extracted the following information using a pre-designed data extraction sheet developed using Microsoft Excel: author name, publication year, study design, sample size, follow-up duration, and baseline characteristics of participants. Two investigators performed a quality assessment for included studies using the Newcastle-Ottawa scale for observational studies and the Cochrane risk of bias assessment tool for RCTs.

Statistical Analysis

Data analysis was performed using Review Manager (version 5.4.1, The Cochrane Collaboration, Oxford, England). Statistical heterogeneity among studies was evaluated through the Cochran chi-square test, coupled with the I2 statistic. If the chi-square test yielded nonsignificant results (P > 0.10) and the I2 statistic was below 50%, indicating a lack of heterogeneity, a fixed-effect model was employed. Conversely, when heterogeneity was present, the random-effect model was utilized for analysis. The outcome variables were compared between the two groups using the risk ratio (RR) with a 95% confidence interval (CI), estimated by the Mantel-Haenszel χ2 method. Significance was determined at P values < 0.05.

Results

**Figure 1:**
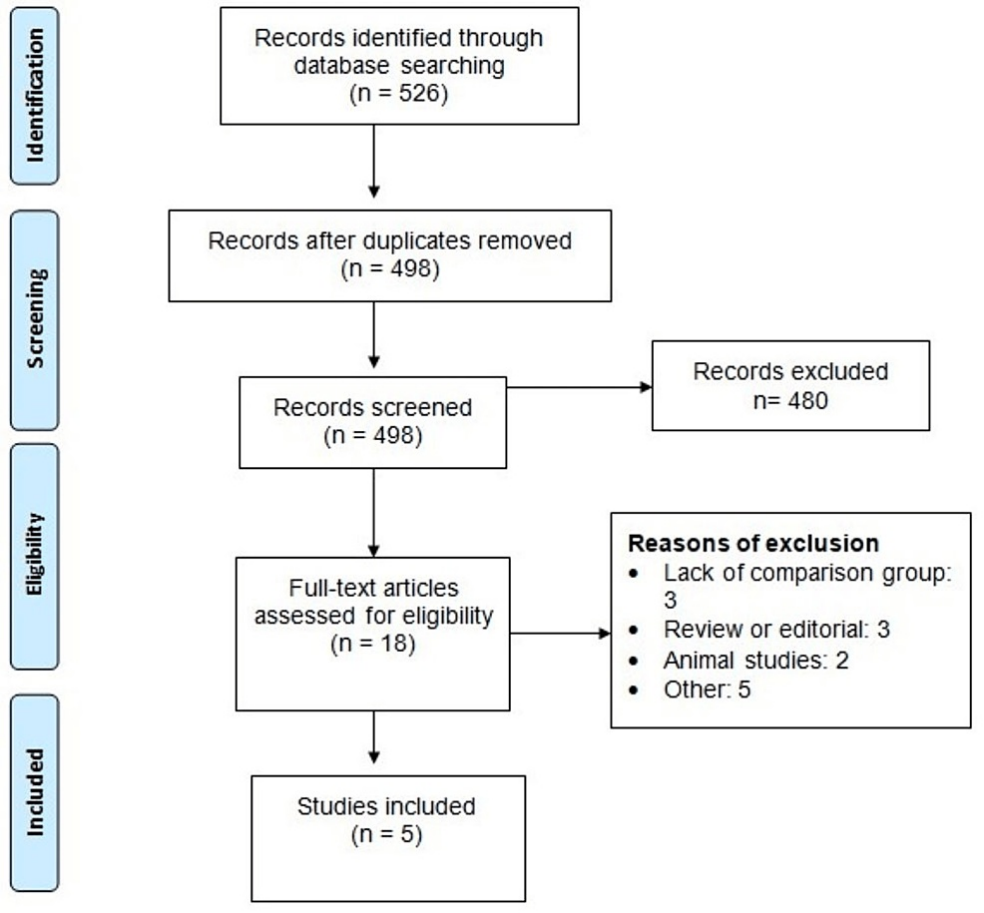
PRISMA flowchart of the study selection

Figure 1 shows the process of study selection. Of 526 articles that were identified from the literature search, 480 studies were excluded after an abstract and title review. Of the 17 articles for further review, five articles were included in the meta-analysis, enrolling a pooled sample size of 876 patients, including 405 in the warfarin monotherapy group and 471 in the warfarin plus aspirin group. Table 1 summarizes the characteristics of the studies included in the present analysis. Among the five studies, four were observational, and one was an RCT. These studies were carried out in various locations, including the United Kingdom, the United States, Italy, and Saudi Arabia. The RCT was a multicentric study conducted across Asia, Europe, Australia, and America. Table 2 presents the quality assessment of the included studies.

**Table 1:** Characteristics of the included studies RCT: Randomized control trial; NR: Not reported

Author	Year	Region	Study Design	Groups	Sample Size	Age (Years)	Males (n)	Diabetes (n)
Lim et al. [9]	2020	United Kingdom	Observational	Warfarin	27	56	23	11
				Warfarin+Aspirin	53	51	42	7
Mehra et al. [10]	2023	Multicenter	RCT	Warfarin	296	58	224	134
				Warfarin+Aspirin	293	57	232	106
Tarzia et al. [11]	2023	Italy	Observational	Warfarin	22	61.5	19	7
				Warfarin+Aspirin	28	63.1	26	14
Tuyl et al. [12]	2017	Saudi Arabia	Observational	Warfarin	32	57	29	8
				Warfarin+Aspirin	44	58	29	19
Yi et al. [13]	2023	United States	Observational	Warfarin	28	NR	NR	NR
				Warfarin+Aspirin	53			

**Table 2:** Quality assessment of the included studies

Quality Assessment for Observational Studies					
Study ID	Selection	Comparability	Outcome	Overall	
Lim et al. [9]	3	2	3	Good	
Tarzia et al. [11]	3	1	2	Fair	
Tuyl et al. [12]	3	2	3	Good	
Yi et al. [13]	4	2	2	Good	
Quality Assessment for Randomized Controlled Trial					
Study ID	Selection	Performance	Attrition	Reporting	Other
Mehra et al. [10]	Low	Low	Low	Unclear	Low

Any Thrombotic Event

**Figure 2:**
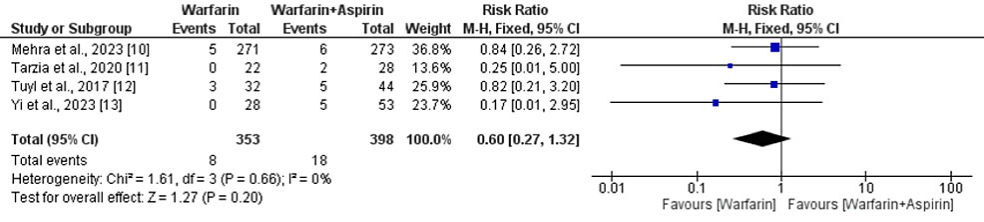
Comparing the risk of thrombotic events between the two groups Sources: References [10-13]

Four studies compared the effect of warfarin alone and warfarin plus aspirin on thrombotic events, and the pooled RR showed that the risk of thrombotic events was not significantly different between the two groups (RR: 0.46, 95% CI: 0.15-1.37), as shown in Figure 2. No significant heterogeneity was reported among the study results (I-square: 0%).

Bleeding Events

**Figure 3:**
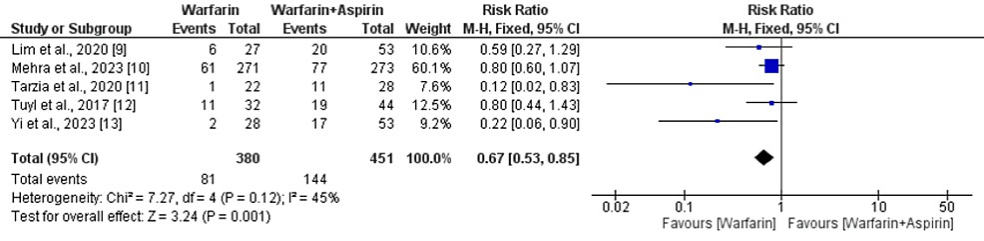
Comparing the risk of bleeding events between the two groups Sources: References [9-13]

A total of five studies compared the effect of warfarin alone and warfarin plus aspirin on bleeding events. The pooled incidence of bleeding events was 27.1%. As shown in Figure 3, the risk of bleeding events was 33% lower in patients receiving warfarin alone compared to patients receiving aspirin plus warfarin (RR: 0.67, 95% CI: 0.53-0.85), and the difference was statistically significant. No significant heterogeneity was reported among the study results (I-square: 45%).

All-Cause Mortality

**Figure 4:**
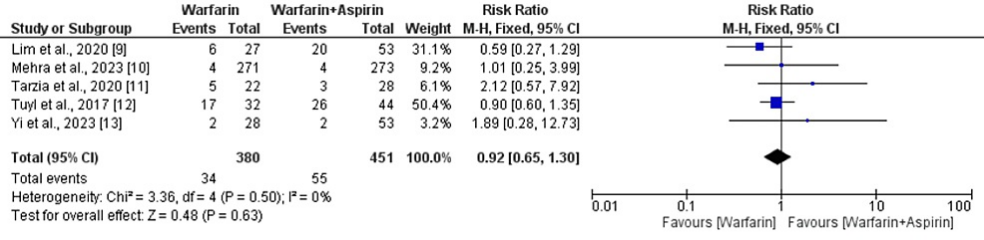
Comparing the risk of death between two groups Sources: References [9-13]

A total of five studies compared the risk of all-cause mortality between the warfarin alone group and the warfarin plus aspirin group. As shown in Figure 4, the risk of all-cause mortality was not significantly different between patients receiving warfarin alone and patients receiving aspirin plus warfarin (RR: 0.92, 95% CI: 0.65-1.30). No significant heterogeneity was reported among the study results (I-square: 0%).

Discussion

The present meta-analysis incorporated five studies that compared the efficacy of warfarin monotherapy with that of warfarin in combination with aspirin in patients with ventricular assist devices. The results of our meta-analysis reveal a significantly reduced risk of bleeding events among patients receiving warfarin monotherapy, without a concurrent increase in thrombotic events and mortality. The combination of antiplatelet agents, such as aspirin, with oral anticoagulants is commonly assumed to enhance thrombotic event prevention beyond the scope of anticoagulant monotherapy in individuals with a continuous flow left ventricular assist device (CF-LVAD). However, findings from both prior research and our current study suggest that aspirin therapy might not be effective in diminishing the risk of thrombotic complications in patients with a HeartMate II (HM II) [14,15]. Furthermore, our study indicates that antithrombotic therapy using a high-dose aspirin of 325 mg daily did not exhibit a reduction in thrombotic complications when compared to treatment strategies involving low-dose aspirin of 81 mg daily [14].

The established standard of care for durable continuous-flow left ventricular assist devices (CF-LVADs) has included the incorporation of aspirin alongside warfarin since their inception, as endorsed by CF-LVAD consensus documents and guidelines dating back to 2010 [16,17]. Antithrombotic therapy approaches for LVADs currently exhibit substantial heterogeneity. A comprehensive literature review has identified varied protocols, encompassing combinations such as warfarin in conjunction with both aspirin (ASA) and clopidogrel therapies, addressing both the immediate post-implant period and chronic support [18]. Typically, a combination of warfarin with a platelet inhibitor is recommended and widely adopted as the primary therapeutic strategy for LVADs to mitigate the risk of thrombosis. Nevertheless, the necessity for additional antiplatelet therapy alongside vitamin K antagonists (VKA) remains a topic of ongoing debate. This debate is exemplified by considerable diversity in antiplatelet therapy regimens observed across different centers and devices, as evidenced by a systematic review encompassing 24 predominantly observational studies [19].

Practice variations likely stem from concerns about bleeding events when combining antiplatelet therapy with VKA, especially in patients prone to acquired von Willebrand syndrome and impaired platelet function. Some studies completely avoid antiplatelet therapy, while others recommend dual antiplatelet therapy with aspirin and dipyridamole or clopidogrel. In patients with axial devices treated with aspirin and dipyridamole, the incidence of thromboembolic events was significantly lower than in those treated with aspirin alone (10% vs. 19%, RR: 0.50; 95% CI: 0.36-0.68) [19]. Corresponding rates of ischemic stroke were 6% and 10%. For patients treated without aspirin, the rate of thromboembolic events was 14%, not exceeding that in aspirin-treated patients (RR: 1.43, 95% CI: 0.81-2.5). However, it is essential to interpret these results cautiously, considering potential imbalances in INR intensity, patient characteristics, and device specifications among the studies. In the European TRACE study, VKA monotherapy was employed in the management of 101 patients with HeartMate II devices [19].

Post-implantation, bleeding events stand out as the predominant reason for readmission after left ventricular assist device (LVAD) placement [20]. The early post-operative period sees bleeding incidents, often necessitating re-operation, occurring in 6%-69% of LVAD patients. Additionally, beyond the immediate post-operative phase, about 30% of patients encounter major bleeding complications. The heightened risk of bleeding is attributed to multiple factors, including the routine use of VKA and antiplatelet therapy, along with the previously mentioned risks of acquired coagulopathies and angiodysplasias [21].

The precise mechanisms leading to angiodysplasia formation in LVAD patients are not fully elucidated. Recent findings suggest a dysregulation of angiopoietin 2, a powerful angiogenic factor stored in endothelial cells within Weibel-Palade bodies, in patients with continuous flow LVADs [22]. In comparison to individuals with heart failure or orthotopic heart transplantation, LVAD patients exhibit higher serum levels and endothelial expression of angiopoietin 2. Elevated angiopoietin 2 levels have been associated with increased angiogenesis in vitro, a phenomenon normalized with angiopoietin-2 blockade. The potential of targeting angiopoietin 2 as a pharmaceutical intervention to prevent bleeding complications in LVAD patients requires further investigation [23].

Crucially, the study conducted by Mehra et al. [10] underscores that the advantages of omitting aspirin remain consistent, even among those with previous vascular diseases such as surgical or percutaneous coronary revascularization, obesity, or diabetes, characteristics traditionally linked to an elevated risk of thrombosis. Hematological changes, such as the development of acquired von Willebrand disease, may reduce the predisposition to thrombosis, and VKA therapy might furnish adequate antithrombotic effects without necessitating concurrent aspirin use. Consequently, discontinuing aspirin while continuing warfarin in LVAD patients poses no compromise and may even be advantageous in lowering the risk of significant nonsurgical bleeding events. However, the uncertainties associated with discontinuing anticoagulation in LVAD patients warrant a case-by-case decision-making approach, as the risks are not well-defined [24].

Study Limitations

The existing meta-analysis has several limitations. Firstly, the inclusion was limited to only five studies, with four of them being observational. Notably, there is only one RCT that directly compares warfarin monotherapy with the combination of warfarin and aspirin in patients with a left ventricular device. To provide more robust guidance for clinicians, it is imperative to conduct future multicenter RCTs comparing these two therapeutic approaches. Secondly, the absence of individual-level data prevented us from conducting subgroup analyses. Additional data are needed to delve into the specific patient populations that might derive distinct benefits from each of the two anticoagulation approaches within this patient cohort.

## Conclusions

In conclusion, our meta-analysis, encompassing five studies and a pooled sample size of 876 patients, underscores that warfarin monotherapy, in comparison to warfarin plus aspirin, significantly reduces the risk of bleeding events in left ventricular assist device (LVAD) patients without an elevation in thrombotic events or mortality. The ongoing debate on the need for antiplatelet therapy alongside vitamin K antagonists is evident in the considerable heterogeneity in current antithrombotic strategies. Despite the potential benefits of discontinuing aspirin, the decision should be approached cautiously, considering the undefined risks of discontinuing anticoagulation in LVAD patients. Future multicenter RCTs and additional data are crucial to further guide clinicians in optimizing anticoagulation strategies for LVAD patients.
